# Advancement in Cancer Stem Cell Biology and Precision Medicine—Review Article Head and Neck Cancer Stem Cell Plasticity and the Tumor Microenvironment

**DOI:** 10.3389/fcell.2021.660210

**Published:** 2022-01-03

**Authors:** Molly E. Heft Neal, J. Chad Brenner, Mark E. P. Prince, Steven B. Chinn

**Affiliations:** ^1^ Department of Otolaryngology-Head and Neck Surgery, University of Michigan, Ann Arbor, MI, United States; ^2^ Rogel Cancer Center, University of Michigan, Ann Arbor, MI, United States

**Keywords:** head and neck cancer, cancer stem cell, tumor microenvironment, niche, heterogeneity

## Abstract

Head and Neck cancer survival has continued to remain around 50% despite treatment advances. It is thought that cancer stem cells play a key role in promoting tumor heterogeneity, treatment resistance, metastasis, and recurrence in solid malignancies including head and neck cancer. Initial studies identified cancer stem cell markers including CD44 and ALDH in head and neck malignancies and found that these cells show aggressive features in both *in vitro* and *in vivo* studies. Recent evidence has now revealed a key role of the tumor microenvironment in maintaining a cancer stem cell niche and promoting cancer stem cell plasticity. There is an increasing focus on identifying and targeting the crosstalk between cancer stem cells and surrounding cells within the tumor microenvironment (TME) as new therapeutic potential, however understanding how CSC maintain a stem-like state is critical to understanding how to therapeutically alter their function. Here we review the current evidence for cancer stem cell plasticity and discuss how interactions with the TME promote the cancer stem cell niche, increase tumor heterogeneity, and play a role in treatment resistance.

## Introduction

Head and neck cancer accounts for approximately 60,000 new cancer diagnoses and 13,000 cancer related deaths in the United States each year. Overall survival for head and neck cancer averages around 50%. Despite advances in treatment modalities these statistics have remained relatively unchanged over the past 30 years (Siegel et al., 2015). Regional and distant metastases remain the leading cause of treatment failure in head and neck cancer patients (Chinn et al., 2015). Cancer stem cells (CSCs) have been theorized to be a leading cause of treatment failure and recurrence. In head and neck tumors, CSCs have been associated with advanced T stage, regional and distant metastases, perineural invasion, radiation failure, and shorter disease-free survival (Wang et al., 2009; Joshua et al., 2012; Chinn et al., 2015).

There are two contrasting, although not mutually exclusive, models of tumorigenesis (Reya et al., 2001; Shackleton et al., 2009). In the clonal model a population of cells gain a proliferative advantage through various mutations and environmental factors that drives tumor growth. In this model the tumor is made up of heterogenous cells that are all capable of creating a new tumor. In contrast, the cancer stem cell model proposes that there exists a limited subset of cells that are capable of regenerating various cell types that make up the tumor and that these progenitor cells are unable to create a *de novo* tumor (Wang and Dick 2005; Dick 2008). Proponents of the cancer stem cell model argue that because CSCs are thought to be relatively resistant to radiation and chemotherapy; they evade initial treatment modalities and subsequently are able to recreate the heterogeneous tumor. As such it is this small subset of cells that needs to be targeted in order to eradicate the tumor (Bao et al., 2006; Dick 2008; Al-Assar et al., 2009; Mroz et al., 2013; Dionne et al., 2015; Subramanian et al., 2017; Zonga et al., 2018; Hu et al., 2019; Shibata and Hoque 2019; Keysar et al., 2020; Saito et al., 2021). More recently there has been an evolution in this model that suggests an inherent plasticity to CSCs that is mediated through the TME, arguing against the unidirectionality of the CSCs models (Chaffer et al., 2013; Sancho et al., 2015; Shimokawa et al., 2017; Ahmed et al., 2018; Yao et al., 2019; Heise and Sommer 2021). This highlights the importance of a deeper understanding of the plasticity and interactions with the TME to identify new ways to target cancer stem cells to move the field forward.

While CSCs were initially characterized in hematologic malignancies, the evidence for their role in solid tumors, including head and neck cancer (HNC), remains robust (Reya et al., 2001; Prince et al., 2007; Chinn et al., 2015; Prince et al., 2016). Multiple cell markers have been identified and utilized to isolate CSCs in HNC. One of the first and most widely cited surface markers for CSCs identified in HNC is CD44 (Škerlová et al., 2015). Initial studies by Prince et al. found that CD44 positive but not CD44 negative cells were capable of regenerating tumors in mouse xenograft models, maintained the ability to be further passaged, and histologically mirrored primitive cells. Additional work by Prince and Chinn et al. revealed increased rates of tumorigenesis, decreased time to regional metastasis, increased rate of metastatic growth, and a higher likelihood of distant metastases in mice with CD44 high tumors verses CD44 low tumors (Prince et al., 2007; Davis et al., 2010; Chinn et al., 2015). Based on work done in other cancers, additional stem cell markers have been identified in HNC (Zhou et al., 2007; Wei et al., 2009; Chen et al., 2011; Martens-de Kemp et al., 2013; Yan et al., 2013; Prince et al., 2016; Fukusumi et al., 2018). Of these, aldehyde dehydrogenase (ALDH) has been a highly specific cancer stem cell marker, specifically when co-analyzed with CD44. It is CD44’s role as a surface protein involved in cell-cell interactions, adhesion, and migration that further supports the mechanistic study of CD44+ stem cells in interaction with the tumor microenvironment (Škerlová et al., 2015; Ferreira et al., 2018; Choi et al., 2021). These data support the clinical importance of CSCs in tumorigenesis, metastasis, and treatment failure.

There is mounting evidence that CSCs require close interactions with neighboring cells within the tumor microenvironment in order to survive and in order to allow for the plasticity inherent to CSCs (Medema and Vermeulen 2011). It is within this ecosystem that the CSC niche is maintained (Borovski et al., 2011; Marusyk et al., 2012; Plaks et al., 2015). A thorough understanding of the crosstalk between CSCs and the tumor microenvironment (TME) that allows for CSC maintenance is a critical step towards the discovery of therapeutic targets in head and neck cancer.

Here we discuss the plasticity of cancer stem cells and impact of the TME, metabolic reprogramming, and potential translational strategies to target head and neck CSCs plasticity as a means for novel therapeutic strategies.

## Cancer Stem Cell Plasticity

As described previously, in contrast to the clonal model, the cancer stem cell model suggests a unidirectional hierarchical process by which CSCs can give rise to progenitor cells, but progenitor cells cannot give rise to CSCs. Recent evidence suggests that there is a fluid state by which CSCs and non-stem cancer cells can interconvert between stem and non-stem like states; thus integrating both the CSC and clonal models (Medema 2013). The work by Chaffer et al. were the first group to demonstrate this “bidirectional interconversion” of breast CSCs and non-CSCs. Here they demonstrated that differentiated transformed and non-transformed human mammary epithelial cells can convert into a “stem-like state” and that this occurs in the absence of new genetic alterations. Furthermore, this ability to interconvert between a non-stem-like and stem-like state in transformed cells occurred at a significantly higher rate than seen in non-transformed cells, further suggesting a unique mechanism for CSC plasticity in cancer (Chaffer et al., 2011). The mechanism of this plasticity in breast CSCs was found to be dependent on activation of ZEB1, a critical mediator of epithelial-mesenchymal transition. Here the non-stem state is maintained by inactivation of ZEB1 through a bivalent chromatin state. Various stimuli then convert the bivalent chromatin into an active form with increased ZEB1 expression in the stem-like state (Chaffer et al., 2013). Several studies have looked at ZEB1 and its interaction with maintenance of a stem state (Yuan et al., 2019; Nong et al., 2021; Pérez et al., 2021). Within head and neck cancer, ZEB1 overexpression was found in cells enriched for CD133 and was associated with increased tumor initiation, again suggesting the importance of ZEB1 and the EMT in the CSC phenotype (Chu et al., 2013). Given an EMT regulator was found to mediate interconversion and maintenance of a stem-like state, this offers a critical area of study to target CSC maintenance and plasticity. Additional work evaluating the impact of CSC interconversion by Gupta et al. further examined the plasticity model using in silico gene set enrichment analysis and mathematical modeling. Here they found breast cancer cells fluctuate between luminal, basal, and stem cell phenotypes to reach an equilibrium state, even after sorting, thus suggesting that breast CSC can arise *de novo* from non-CSC. This study further demonstrated that sensitivity to systemic chemotherapy is dependent upon the phenotypic state of the tumor cells, further illustrating the critical need to understand plasticity for therapeutic use (Gupta et al., 2011).

Despite this seminal work, only a few studies have explored CSC plasticity, mechanism, and potential targeting for therapy: with even fewer in head and neck cancer. A recent study by Leong et al. suggests that HNC cell lines also share a similar propensity to return to an equilibrium state. HNC cell lines enriched for ALDH showed that ALDH+ and ALDH- populations would return to a steady state of admixed CSCs and non-stem CSC (Leong et al., 2014). This interconversion was found to be driven in part by EGFR and IGF-1R pathway activation. Xie et al. evaluated targeting differentiation pathways in nasopharyngeal cancer as a means of altering stem transitions and impacting cell survival (Xie et al., 2021). These studies suggest that the rigid unidirectional aspect of the initial CSC theory may not be the whole story. This has important implications in treatment paradigms, as the goal of targeting CSCs may yield initial eradication of the CSCs however if progenitor cells possess the ability to convert into CSCs this may lead to ultimate treatment failure. Whether the conversion between CSCs and non-stem can cells is driven by environmental factors, genetic alteration, or a stochastic process is still debated (Morel et al., 2008; Medema 2013) however these results suggests that targeting the mediating factors that control the interconversion and maintenance of the CSC niche offers a potential therapeutic option.

## Cancer Stem Cell Reprogramming

Metabolic reprogramming is thought to play a role in both tumor formation by CSCs and in the plasticity of CSCs (Menendez et al., 2013). Metabolic signaling (whether it be epigenetic changes or environmental interactions) can affect the rate at which CSCs and non-stem cancer cells interconvert and can thereby dictate the tumorigenicity. As described by Menendez et al. these metabolic factors can be thought of as “facilitators” or “impediments” in this interconversion. Initial advances in this area involved the discovery of metabolic signaling that induced pluripotent stem cells from somatic cells (Hanna et al., 2010), which involves expression of known CSC markers such as Oct4 and Sox2 (Carey et al., 2011). This idea was subsequently applied to development of stem cells in cancer. While not the only factor necessary for development of CSCs, certain metabolic environments will increase the likelihood of the epigenetic and ultimately stochastic events that lead to interconversion of non-stem cancer cells (Menendez et al., 2013). Proposed metabolic reprogramming events include the propensity for tumor cells to switch to anaerobic glycolysis, one of the initial tenants of cancer suggested by Warburg (Warburg 1956). This has been shown in breast, osteosarcoma and nasopharyngeal CSCs (Cioce et al., 2014; Palorini et al., 2014; Shen et al., 2015), and inhibition of this metabolic reprogramming reduced stemness in nasopharyngeal cancer cells. Anaerobic glycolysis is further supported by CSCs dependency on HIF-1α (Gammon et al., 2013; Nam et al., 2016). Liu et al. looked at the mechanism of tumor associated fibroblasts (TAFs) on maintenance of a stem-like state and increased glycolysis through regulation of HIF-1α (Liu Y. et al., 2021). Here, they identified a specific microRNA (miR-7641) that could silence HIF-1a expression and alter breast cancer cells glycolysis ability and stem cell gene expression. This alteration of the metabolic pathway through paracrine interaction of the supporting stromal cells further supports the critical impact of the TME on stem cell maintenance. This interaction of an oxidative state and metabolic reprogramming for CSC maintenance is further supported based on the shift towards hypoxia and increased reactive oxygen species (further elucidated below), or by overexpression of inflammatory markers such as cyclooxygenase-2 (COX-2) (Tian et al., 2017). Epigenetic and post-translational alterations provide additional mechanisms for cancer stem cell reprogramming. Dong et al. demonstrated the importance of super enhancers in promoting cancer stem cells (Dong et al., 2021) and there is mounting evidence for the role of microRNAs in cancer stem call regulation (Bourguignon et al., 2012; Barlak et al., 2020; Fitriana et al., 2021). These necessary metabolic and other perturbations are often driven by interactions with neighboring cells in the tumor microenvironment. However, despite burgeoning research in this area, only a single study to date has evaluated the role of metabolic reprogramming in head and neck CSC and this was solely in nasopharyngeal cancer (Shen et al., 2015), thus representing a novel area of study. Despite limited data in metabolic reprogramming, there has been significant data evaluating the stromal maintenance of CSC.

## The Perivascular Niche

Evidence has shown that endothelial cells are influenced by neighboring cancer cells to create a perivascular niche. Initially discovered in neural tumors (Shen et al., 2004; Calabrese et al., 2007), this idea of a perivascular niche has more recently been described in head and neck tumors as well as other solid malignancies. Studies have shown that cancer stem cells reside within these specialized areas (Krishnamurthy et al., 2010; Ghajar et al., 2013). Krishnamurthy et al. utilized patient derived HNC cells implanted into xenograft mouse models to demonstrate the tumor generating potential of CD44+/ALDH+ tumor cells compared to CD44-/ALDH- tumor cells. In this experiment they also implanted human endothelial cells to allow for neovascularization surrounding the tumors. As expected the CD44+/ALDH+ cells resulted in a significantly higher number of tumors after implantation compared to the CD44−/ALDH− tumors cells. Histologic analysis of these tumors revealed that the majority of ALDH+ stem like tumor cells were localized within 100 um of blood vessels. Subsequent histologic analysis of primary head and neck oral cavity tumors revealed that approximately 80% of stem like cancer cells (defined as ALDH+) were located within 100 um of blood vessels. To further evaluate the role of endothelial cells in creating a perivascular niche, CSCs and non-stem cancer cells were grown in the presence of endothelial cell conditioned medium. There was a significant increase in sphere formation in the CD44+/ALDH+ cells in the presence of the conditioned media. Further, conditioned media resulted in increased expression of BMI -1 suggesting a role of endothelial cells in promoting CSCs potential for epithelial-to-mesenchymal transition (EMT) and metastasis. Validation experiments also demonstrated that ablation of endothelial vessels resulted in a decreased number of CSCs in xenograft models. A second study by this same group further evaluated the role of endothelial cells on maintenance of CSCs and aimed to identify the factors that regulate the CSCs niche. This study identified that IL-6 secreted by endothelial cells promoted tumorigenesis and survival of CSCs, and that inhibition of IL-6 through shRNA or treatment with an anti-IL-6 antibody (tocilizumab) reduced this effect (Krishnamurthy et al., 2014). These two studies support the role of endothelial cells in maintaining CSCs in head and neck tumors and suggest that targeting endothelial cells within the tumor may also affect CSCs residing within the perivascular niche.

**FIGURE 1:**
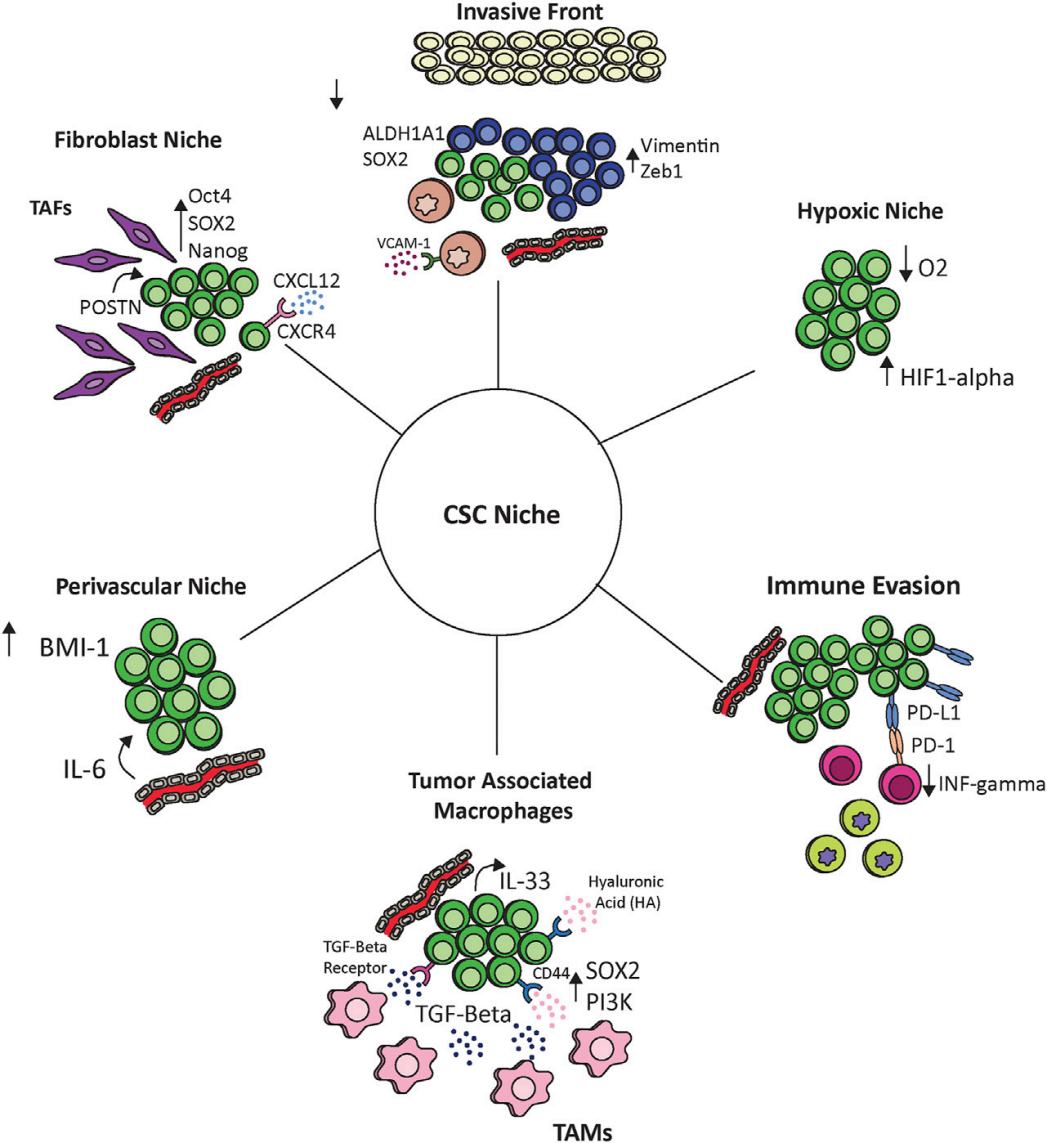
The interaction of cancer stem cells with the tumor microenvironment. Cancer stem cells communicate with and react to the tumor ecosystem including endothelial cells, hypoxic environments, tumor associated fibroblasts (TAFs), tumor associated macrophages (TAMs), monocytes, and other immune cells. These interactions maintain the cancer stem cell niche and provide potential therapeutic options.

In contrast to the perivascular niche, which remains a well-oxygenated environment due to its proximity to blood vessels, there is also evidence that CSCs are maintained through hypoxic conditions (Ritchie and Nor 2013). Alluding back to the idea of metabolic reprograming, A study by Wu et al. demonstrated increased percentages of CSCs (defined by CD133) and increased expression of stem cell markers including NANOG, SOX2, and OCT4 in laryngeal tumor cell lines exposed to hypoxic conditions (Wu et al., 2014). Whether hypoxia promotes CSCs growth through direct mechanisms or selects for CSCs due to death of non-stem cancer cells is less clear. A study by Duarte et al. demonstrates that hypoxic conditions resulted in an overall decrease in growth of HNC cell line models and that the proportion of CSCs in the surviving group increased potentially suggesting that hypoxia selects for CSCs as a survival mechanism in adverse conditions (Duarte et al., 2012). Furthermore, a study by Marcu et al. utilizing in silica models suggests that there is increased resistance of CSCs to radiation therapy when exposed to hypoxic conditions (Marcu et al., 2016). Cancer stem cells residing within hypoxic niches contribute to the overall resistance of HNC to radiation therapy and additional evidence suggests this may be mediated through a Hif-1 alpha mediated mechanism (Wozny et al., 2017). Together these studies demonstrate the plasticity of CSCs in their ability for survival and tumor maintenance in varying conditions (Figure 1).

## Stromal Niche and Tumor Associated Fibroblasts

Fibroblasts residing within the tumor stroma are also thought to play a critical role in maintenance of the tumor microenvironment and in tumorigenesis (Xing et al., 2010; Wheeler et al., 2014). These tumor associated fibroblasts (TAFs) are characterized by high levels of smooth muscle actin (alpha SMA), fibroblast activation protein (FAP), Thy-1, desmin, and S100A4 protein expression (Garin-Chesa et al., 1990). In head and neck squamous cell carcinoma, TAFs have been shown to promote tumor invasion and are associated with disease progression and worse survival outcomes (Lin et al., 2011; Wheeler et al., 2014). Studies have aimed to identify pathways by which TAFs promote tumor growth. Select pathways have been identified including TGF-Beta1 and EGFR mediated mechanisms (Marsh et al., 2011; Magan et al., 2020). More recent studies have now discovered significant crosstalk between cancer stem cells and TAFs within the TME and data suggests this may play a previously unrecognized role in CSCs maintenance and tumor progression (Yu et al., 2018; Liu et al., 2020). One of these mechanistic studies by Yu et al. which evaluated the effect of TAF secreted periostin (POSTN) on HNC, and found that HNC tumor cells treated with POSTN showed increased expression of stem cell markers including CD166, SALL4, CD271, CD90, CD133, OCT-4, ALDH, SOX2, and NANOG as well as increased spheroid formation (Yu et al., 2018). They also showed that knockdown of protein tyrosine kinase 7 (PTK7) dampened this response, suggesting a mechanistic dependence on the POSTN-PKT7 axis. Further experiments using both *in vitro* and *in vivo* models demonstrated that activation of this axis not only increased tumorigenicity but also increased activation of the wnt/B-Catenin pathway. This study is one of the first to define a mechanistic pathway by which TAFs maintain the CSCs niche. The CXCR4-CXCL12 axis has also been implicated as a stromal mediator of the CSC niche. Best described in hematologic malignancies, this axis has been shown to play a key role in numerous solid malignancies such as breast, esophageal, and pancreatic cancer among others and is of particular interest as there are multiple small molecule inhibitors of CXCR4 and CXCL12 that are under investigation (Hermann et al., 2007; Popple et al., 2012; Wang et al., 2017; López-Gil et al., 2021). In head and neck cancer there are mixed studies investigating this pathway. Faber et al. found that CXCR4 expressing cells were highly expressed in tumor nests but did not co-localize to the stroma with the CD44 expressing cells (Faber et al., 2013a). In contrast to these results, the same group also demonstrated that in the head and neck cancer cell line UMSCC-11A CD44+/CXCR4+ cells showed increased podia formation with the additional of CXCL12 suggesting a role of this axis in the regulation of CD44+ CSC (Faber et al., 2013b). A study by Jungbauer et al. further evaluated the role of CXCL12 on HPV+ and HPV− head and neck cancer cell lines and found that only HPV− cells lines showed increased podia formation in response to CXCL12 (Jungbauer et al., 2017). Together these results suggest the CXCR4/CXCL12 axis as a promising area of further research.

## CSCs and Tumor Associated Macrophages

Immune cells additionally play a key role in maintaining the TME and creating an immunosuppressive milieu. Tumor associated macrophages (TAMs) are thought to closely mirror the subset of M2 macrophages which promote wound healing and have pro-tumorigenic properties (Chanmee et al., 2014; Aras and Zaidi 2017; Mantovani et al., 2017). Associations between TAMs and CSCs has been demonstrated in other solid malignancies such as breast, lung, and colorectal (Rao et al., 2013; Yang et al., 2019). Zhang et al. found that polarized M2 macrophages when co-cultured with lung adenocarcinoma cell lines lead to increased proliferation and stemness. While some evidence in HNC suggests an association between TAMs and CSC in HNC, there is a gap in understanding a clear mechanistic pathway (He et al., 2014; Hu et al., 2016). A recent study by Gomez et al. utilized *in vitro* and *in vivo* models to demonstrate the intricate interactions between TAMs and head and neck CSCs (Gomez et al., 2020). In this study head and neck cancer cells were co-cultured with macrophages to mimic an established TME which resulted in increased levels of PI3K-4EBP1 phosphorylation, SOX2, and ALDH1A1, compared to HNC cells cultured alone. There was also a higher proportion of CD44+/ALDH high cells in the co-culture group indicating increased stemness of the population. Conversely when HNC cells were co-cultured with immature monocytes, mimicking the leading edge of tumors, there was downregulation of PI3K-4EBP1 phosphorylation, SOX2 and ALDH1A1 and a decreased proportion of CD44+/ALDH high cells. Co-culture with monocytes leads to increased levels of vimentin and Zeb1, both of which are involved in EMT. The authors also found that binding of CD44 to hyaluronic acid (a protein expressed in high levels in the extracellular matrix) resulted in a positive feedback loop that promoted expression of PI3K and SOX2. These results support similar findings from previous studies (Liu and Cheng 2017; Passi et al., 2019). These data also suggest that this positive feedback loop was promoted in the presence of TAMs. Finally, in the models of the invasive front the authors showed that monocyte binding to VCAM-1 increased invasion with associated decrease in CSCs population. Overall this study supports an intimate interaction between TAMs and CSCs and demonstrates how macrophages and monocytes promote a transition between the invasive verses growth phenotypes in CSCs. These results further reflect the role of the TME and CSCs in maintaining tumor heterogeneity. An additional study by Taniguchi et al. also supports the role of TAMs in cancer stem cell maintenance (Taniguchi et al., 2020). In this study authors leveraged a mouse model of squamous cell carcinoma and human squamous cell tumor samples to demonstrate that TAMs create a TGF-Beta rich environment that stimulates CSCs to release IL-33. These paracrine signals then result in further differentiation of immature immune cells into FcεRIα+ tumor associated macrophages. These data support a method of cross-communication between CSCs and cells within the microenvironment that stimulate ongoing tumor growth.

## CSCs and Immune Evasion

In addition to interactions between CSCs and neighboring cells within the TME, there is also evidence that CSCs interact with host immune cells and play a key role in tumor immune evasion (Qi et al., 2012; Maccalli et al., 2018).

Numerous studies have aimed to further delineate the role of CSCs in immune escape. A study by Lee et al. utilizing PDX mouse models inoculated with human tumor cells found that CD44+ cells expressed higher levels of EMT markers and Programmed death-ligand 1 (PD-L1) compared to CD44− cells (Lee et al., 2016). The authors also examined 21 primary tumors and found that CD44+ cells expressed higher levels of PD-L1 at the RNA and protein level. CD44+ cells were also found to induce less INF-gamma expression in CD8+ tumor infiltrating lymphocytes (TILs) when co-cultured with these cells compared to the CD44− subgroups, suggesting lower immunogenicity of the CD44+ population. Interferon gamma expression in these CD44+ cells was rescued with inhibition of the PD-L1/PD1 axis. This effect was not observed in the CD44− subgroup further supporting the interaction between PD-L1 and CD44+ CSCs in the tumor microenvironment. Similar findings of increased PD-L1 expression has also been demonstrated in ALDH+ oropharynx tumor cells compared to ALDH− cells (Tsai et al., 2017). This difference in PD-L1 expression was enhanced after radiation therapy. This same study also correlated ALDH positive tumors with increased levels of peripheral myeloid-derived suppressor cell (MDSC), cells known to play a key role in immune escape.

While decreased major histocompatibility complex (MHC) class 1 expression is a well described mechanism of immune escape in head and neck cancer (Meissner et al., 2005), multiple studies have failed to show differences in MHC class I expression on CSCs compared to paired non-stem cancer cell (Chikamatsu et al., 2011; Liao et al., 2013). However, a study by Chikamatsu et al. did identify possible mechanism by which HNC CSCs exploit the antigen processing and presentation pathway to aid in immune evasion. Type 2 transporter associated with antigen processing (TAP2) is a protein critical for peptide translocation from the cytosol to the lumen of the endoplasmic reticulum, which is required for subsequent peptide loading onto MHC class I proteins. In this study the authors demonstrated a significant difference in expression between CD44+ and CD44− groups, with CD44+ cells having decreased expression of TAP2. Therefore, these data support a possible second mechanism of immune escape that involves suppression of the neoantigen presentation pathway. CD44+ cells also showed increased levels of immunosuppressive cytokines such as IL-8 G-CSF and TGF-Beta when compared to CD44− cells, and showed greater suppression of T cells in culture. Further peripheral blood mononuclear cells co-cultured with CD44+ cells showed increased levels of T regulatory cells and MDSCs, increased IL-10 secretion from peripheral blood monocytes, and increased inhibition of IFN-gamma and IL-2 compared to CD44− negative cells. Together these studies indicate that CD44+ cells promote immune suppression through interaction with host immune cells and suggest a role for immunotherapy as a target for CSCs in HNC (Canter et al., 2016). An important caveat to this, however, is that while inhibition of the PD-1/PD-L1 axis may inhibit one mechanism of immune escape, the secondary escape mechanism of reduced TAP2 protein may render CSCs resistant to monotherapy with anti-PD-1/PD-L1 treatments.

## Targeting Cancer Stem Cells

**FIGURE 2:**
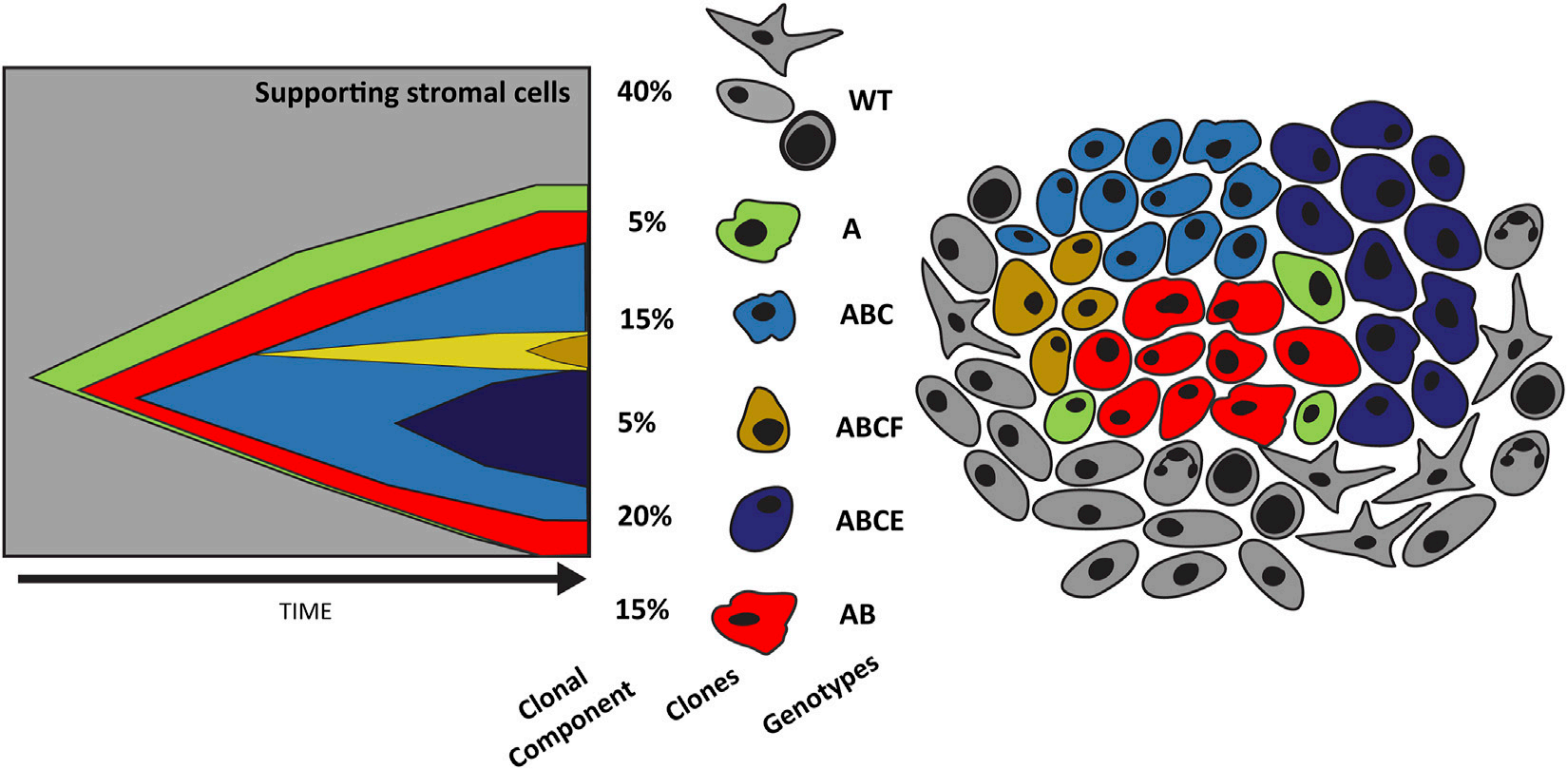
Evolution of tumor heterogeneity. The graph on the left depicts the evolution of cancer cells as described by the original cancer stem cell theory. Here cancer stem cells (green) can replicate and differentiate into individual clones (red) that over time accumulate mutations secondary to selective pressures (blue and yellow). This results in significant tumor heterogeneity as depicted in the illustration on the right. Tumor heterogeneity increases treatment challenges and nominates a conserved population such as cancer stem cells as a potential therapeutic target.

Tumor heterogeneity remains one of the persistent challenges in developing novel cancer therapeutics. As discussed above, cancer stem cells are thought to play a critical role in development of tumor heterogeneity (Sottoriva et al., 2010) and is often cited as a primary mechanism of treatment failure (Greaves 2015). Cancer’s ability to evade both intrinsic (immune response) and extrinsic (cancer therapies) pressures is based on clonal and sub-clonal evolution leading to highly adaptable cellular heterogeneity (McGranahan et al., 2016; Ayob and Ramasamy 2018). One of the challenges of current and novel targeted cancer therapy is the vast burden of molecular data and deciphering of the complex interaction of supporting cells within the tumor ecosystem (Agrawal et al., 2011; Stransky et al., 2011; TCGA 2015). As clonal mutations propagate, selective pressures force evolution of resistant genotypes and phenotypes (Figure 2). Despite significant advances in high-throughput sequencing, heterogeneity may cause critical data points to be missed or averaged out if they are not dominant (Shah et al., 2009; Ding et al., 2010). Recent work in single cell analyses of HNC further demonstrated the extreme diversity of molecular signatures amongst both malignant and non-malignant cells as well as inter- and intra-tumoral heterogeneity (Puram et al., 2017). Deciphering and narrowing the focus to only the critical mediators of tumor heterogeneity is critical to understanding and treating cancer resistance. Given their central importance in hierarchical tumor heterogeneity, CSCs are a highly attractive target for cancer therapy; they likely represent a conserved population that may be more homogenous than the general cancer milieu. However, given their rarity and plasticity, characterizing cancer stem cell signatures with high fidelity remains challenging, yet a crucial step to reduce tumor heterogeneity. Further, given recent advances in discovery of tumor plasticity and the challenges of standard therapeutics in eradicating a heterogenous tumor, targeting the metabolic and immune signals that promote conversion to CSCs may serve as opportunity for novel treatment development.

Recent studies have aimed to develop therapies targeting cancer stem cell plasticity and reprogramming by interfering with the interactions between CSCs and the TME. In head and neck cancer Leong et al. demonstrated that inhibition of EGFR and IGF-1R reduced levels of ALDH+ cells in the population. The authors postulated that this may prove to be an effective strategy for blunting CSC plasticity in the future (Leong et al., 2014). A study by Lee et al. discovered that isoorientin inhibits stemness in oral cavity cell lines through inhibition of the STAT3/Wnt/β-catenin axis and that these results were synergistic in combination with cisplatin (Liu S.-C. et al., 2021). As discussed above, COX-2 has been shown to promote maintenance of CSCs. A study investigating the effects of COX-2 inhibition in hypopharyngeal cancer resulted in deceased expression of genes associated with CSCs and reduced sphere formation. Tumor viability was also decreased and these results were improved by the addition of docetaxel (Saito et al., 2021). Dong et al. demonstrated that inhibition of Bromo- and Extra-Terminal domain (BET) results in an unanticipated impairment of super enhancers and reduced stemness in HNC (Dong et al., 2021). These results suggest that reduction in the cancer stem cell population may reduce tumor heterogeneity and serve as adjunctive therapy to enhance standard therapeutics. Despite the breadth of studies investigating potential interactions between the TME and CSCs, there remains a need for further therapeutic studies targeting CSC plasticity and interruption of the CSC niche.

## Conclusion

There is increasing evidence for the role of CSCs in treatment resistance and recurrence in HNC as well as the role of the TME in maintaining the CSC niche. Here we review an updated model of CSC plasticity and identify potential therapeutic targets including ZEB1, EGFR, and IGF-1R. We also evaluate the evidence for the role of endothelial cells (per-vascular nice), TAFs, and TAMs in promoting CSC growth and maintenance, suggesting another area for therapeutic potential. We also review the critical relationship between CSCs and the host immune system that promotes tumor immune evasion suggesting a potential use of immunotherapy in modulating the CSC niche. Future studies to target these interactions and reduce CSCs plasticity may yield novel therapeutic combinations for HNC as well as other solid malignancies.
